# The Right Internal Jugular at the Cricoid Cartilage Level May Represent the Optimal Central Vein Puncture Site in Pediatric Patients

**DOI:** 10.3389/fped.2022.833845

**Published:** 2022-02-22

**Authors:** Jun Xiong, Huijun Wang, Yun Zhu, Yafen Zhou, Yanan Pang, Liwei Zhang

**Affiliations:** ^1^Department of Anesthesiology, Shenzhen University General Hospital, Shenzhen University, Shenzhen, China; ^2^Department of Anesthesiology, Beijing Tongren Hospital, Capital Medical University, Beijing, China; ^3^Department of Anesthesiology, Sanbo Brain Hospital, Capital Medical University, Beijing, China

**Keywords:** internal jugular vein, ultrasound, cricoid cartilage, thyroid cartilage, pediatric, cross-section area

## Abstract

**Objective:**

Internal jugular vein puncture or cannulation is far more difficult in children compared with adults. Anthropometric measures of the internal jugular vein acquired by two-dimensional ultrasound are useful in the practice of puncture and catheterization. The aim of this study is to measure anthropometric parameters of bilateral internal jugular veins in children and to determine the best puncture site based on these parameters.

**Materials:**

A total of 107 pediatric patients undergoing elective operation were included. Ultrasound-visible evaluation of bilateral internal jugular veins was used to obtain the depth from skin, maximum antero-posterior diameter, and cross-sectional area at the levels of the superior border of thyroid cartilage and cricoid cartilage. Statistical analysis was performed using these anthropometric data and demographic variables of all studied pediatric patients, such as age, height, and weight.

**Results:**

A very weak correlation was noted between the depth, maximal antero-posterior diameter, and cross-sectional area of both internal jugular veins and the age, height, weight, and body surface index of all included children. All Pearson's *R* correlation coefficients were <0.45. The largest diameter and cross-sectional area were in the right internal jugular vein at the cricoid cartilage level (*p* < 0.01) followed by the left internal jugular vein at this level (*p* < 0.01). In addition, the internal jugular vein at the cricoid cartilage level was more superficial than that of the superior border of the thyroid cartilage (*p* < 0.01).

**Conclusion:**

The right internal jugular vein at the cricoid cartilage level is the best site for puncture. The most appropriate alternative site is the left internal jugular vein on the same level. Better correlation was not observed between the anthropometric parameters of the internal jugular vein and children's biological characteristics. This finding should be confirmed in a larger-scale demographical study in the future.

## Introduction

Central venous access is important for diagnostic and therapeutic interventions in pediatric patients who have limited or difficult peripheral venous access. In addition, frequently obtaining peripheral venous access obviously places extra stress on children and their guardians both physically and mentally. For anesthesiologists, temporary central venous catheters are feasible to deliver intravenous medications and continue administration (1). However, central venous cannulation, which is frequently performed through the subclavian vein and internal jugular vein (IJV), is a challenging procedure in children (2). Prior to the use ultrasonography guidance for central venous catheterization, the prevailing technique for this procedure is based on locations of different anatomical landmarks and not direct visualization of the target vein and its ambient structures. Given anatomical differences in children and adults and because veins of pediatric patients are much smaller and more mobile than those of adults (3), they are at higher risk of maneuvering-related complications (4). With the development of ultrasound (US) techniques and devices, US visualization guidance is commonly recommended in central venous catheter placement, especially in pediatric patients (5), given its reduction in complications and improvement in the success rate. With the assistance of US visualization, although practitioners are able to obtain panorama of the IJV, localization of the correct puncture site and catheter placement remain particularly challenging in children because cervical anatomical structures are considerably complex. As an example, a wonderful building is located in front of us. Although we could enter the building through its windows, the most appropriate entrance to the building was its doors.

Several trials have focused on accessing the IJV from the cricoid cartilage (CC) to the subclavian vein in children; therefore, this study demonstrated anatomical variables of the IJV at the level of the CC and superior border of thyroid cartilage (SBTC) with vascular US measurements to identify the best location for IJV puncture and catheterization.

## Patients and Methods

This observational trial was registered prior to patient enrollment with the Chinese Clinical Trial Registry (ChiCTR2100042505, January 22, 2021, Jun Xiong). This study was performed at the tertiary care academic teaching hospital from March to June 2021. The study protocol was reviewed and approved by the Ethical Committee of this hospital. Written informed consent was obtained from legal guardians of all pediatric patients.

A total of 107 pediatric patients aged 6–173 months who required elective operation with general anesthesia were approached for inclusion in this study. Children with serious organ dysfunction, cervical vessel malformation and angioma, high intracranial pressure, superior vena cave syndrome and thrombus, and preoperative shock were excluded.

SonoScape US device (E3, Shenzhen, China) equipment with a high-frequency linear transducer (10I2, 4.0–16.0 MHz) was used. The measuring depth was 3 cm with vascular configuration.

**Figure 1:**
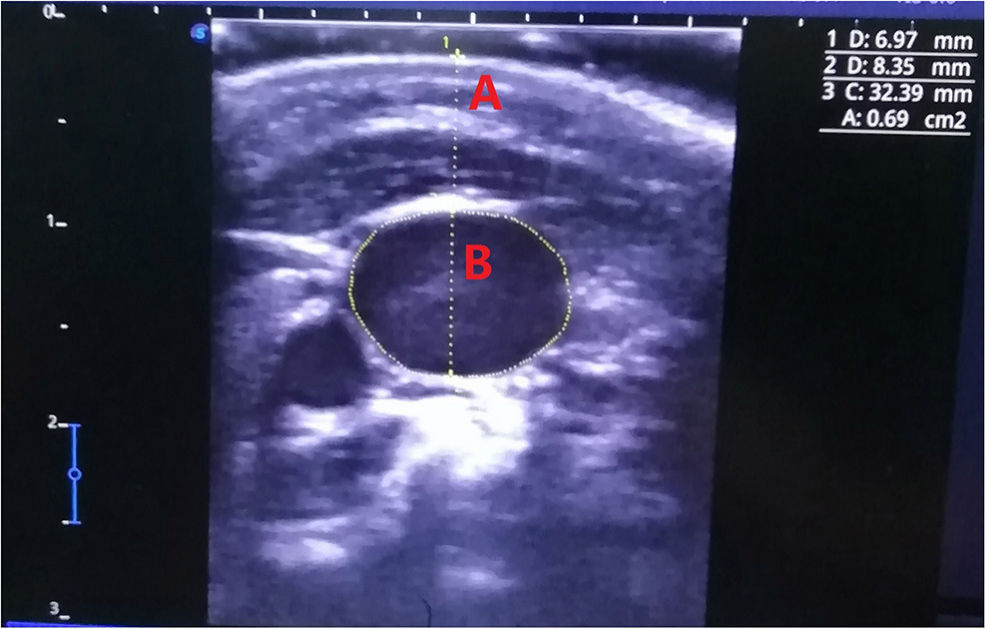
Ultrasound measurement of internal jugular veins.

After general anesthesia induction and intubation, all pediatric patients were placed in the supine 15° Trendelenburg position (6), and their heads were simultaneously rotated 15° contralateral to the examined cervical region with clinical stability (7–9). An arcuate goniometer was used to ensure the degree of head rotation, which was expressed as the rotation degree of nasal bone between eyes. The US probe was located at the SBCT and CC levels to measure the depth, maximum antero-posterior diameter (MAPD), and cross-sectional area (CSA) of the bilateral IJVs on the transverse axis (Figure 1). These anthropometric variables were measured at the end of exhalation. For more accurate assessment of the CSA, the mean of the three measured values of the CSA was calculated. To reduce the deformation by US probe compression as much as possible, the conductive medium between the transducer and skin should be slightly thicker to keep them separated without imaging obstruction. The procedure was performed exclusively by one senior anesthesiologist with abundant US experience.

Pediatric patients' sex, age, weight, and size were recorded. Age was expressed in months, size in centimeters, and weight in kilograms. These parameters were measured by the same health care team. Their body surface area (BSA) was autocalculated by the monitoring system (GE CARESCAPE Monitor B650, Helsinki, Finland) when these demographic variables were input.

All statistical analyses were performed with IBM SPSS Statistics V.21.0 (IBM Corp., Beijing, China). Continuous numerical variables are expressed as the mean and standard deviation and were assessed for normality with the Kolmogorov-Smirnov test and histogram. The means of continuous variables were compared with one-way ANOVA. Homogeneity of variance was evaluated using the Levene test. If equal variances were assumed, then means between groups were compared with *LSD*; otherwise, Dunnett's test was used. Qualitative variables are presented as frequencies and percentages. Correlative analysis and multiple regression models were calculated to estimate the depth, MAPD, and CSA of IJV on the basis of age, size, weight, and BSA. A *p*-value <0.05 was considered statistically significant.

## Results

The open trial included 107 pediatric patients (61.7% boys and 38.3% girls), including children under 1 year old (2.8%), 55 between 1 and 6 years old (51.4%), and 49 older than 6 years of age (45.8%). The demographic data of the pediatric patients are summarized in Table 1. These data were also presented on the basis of the following age distributions: <12 months, between 12 and 72 months, and older than 72 months.

**Table 1:** The characteristics of all patients (61.7% boys and 38.3% girls), and these data were classified by months.

**Classification**	**Variables**	**Mean**	**SD**	**Minimum**	**Maximum**
All	Age (months)	69.4	37.2	1.0	173.0
(107)	Size (cm)	112.4	21.5	50.0	165.0
	Weight (kg)	22.2	8.8	5.0	53.0
	BSA (m^2^)	0.8	0.2	0.3	1.6
<12 months	Age (months)	5.3	4.0	1.0	9.0
(3)	Size (cm)	63.7	7.8	55.0	77.0
	Weight (kg)	6.8	1.6	5.0	8.0
	BSA (m^2^)	0.3	0.1	0.3	0.4
12–72 months	Age (months)	42.3	15.2	13.0	71.0
(55)	Size (cm)	101.0	15.7	50.0	132.0
	Weight (kg)	17.1	4.2	9.5	30.0
	BSA (m^2^)	0.7	0.1	0.3	1.0
>72 months	Age (months)	103.69	22.4	77.0	173.0
(49)	Size (cm)	128.31	13.6	10.0	165.0
	Weight (kg)	28.92	7.8	18.0	53.0
	BSA(m^2^)	1.00	0.2	0.7	1.6

*BSA, body surface index*.

**Figure 2:**
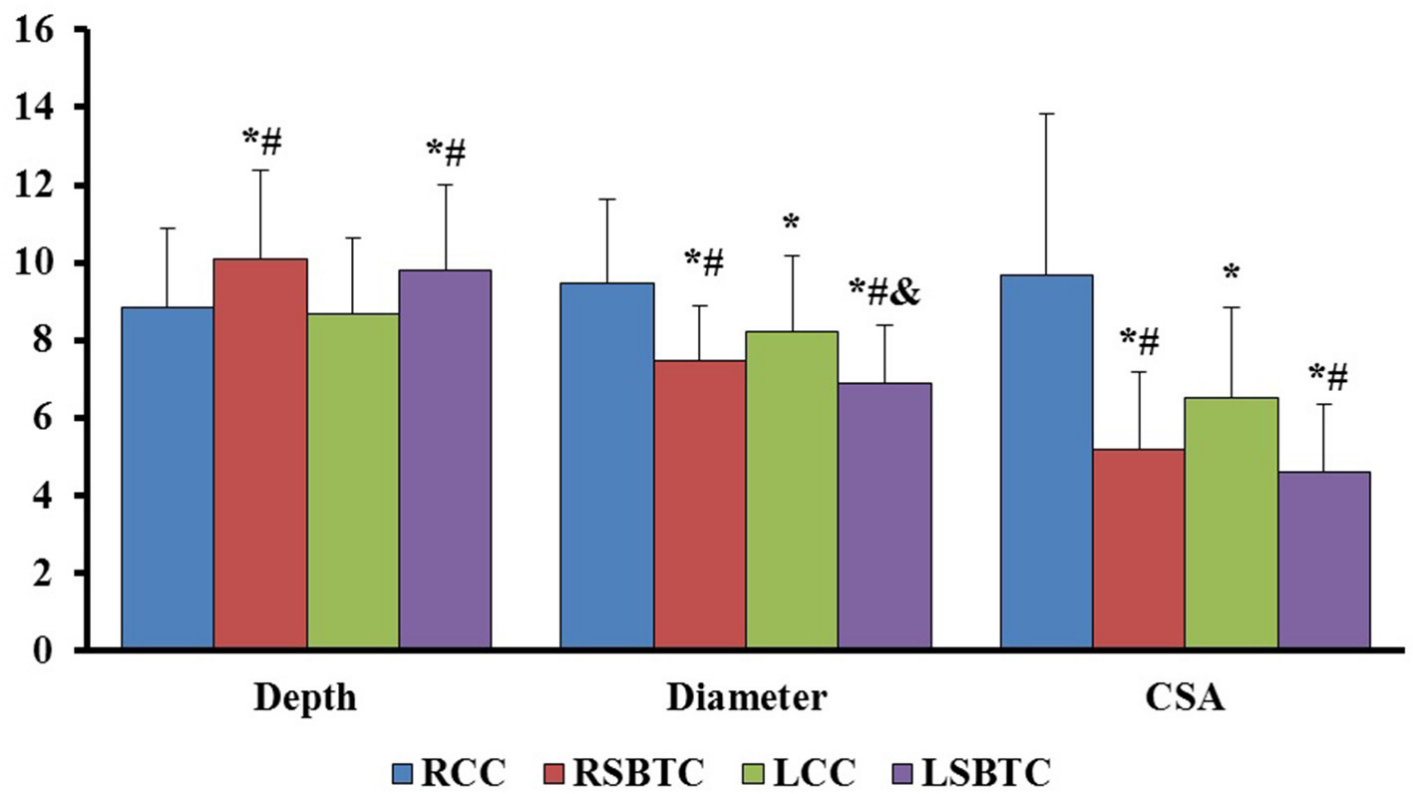
The comparison of depth, MAPD, and CSA of bilateral internal jugular veins. RCC, right cricoid cartilage; RSBTC, right superior border of thyroid cartilage; LCC, left cricoid cartilage; LSBTC, left superior border of thyroid cartilage; MAPD, maximal antero-posterior diameter; CSA, cross-sectional area. The values of CSA were 10 times of realistic CSA measurements. *P < 0.01, compared with RCC; ^#^*P* < 0.01, compared with LCC; ^&^*P* < 0.01, compared with RSBTC.

The depth, MAPD, and CSA of the IJV were acquired on both sides of the neck and at two different levels, namely, SBTC and CC. The detailed parameters are presented in Table 2. At the SBTC level, the bilateral depths of the IJV were similar (*p* = 0.327). At the CC level, the bilateral depths were also similar (*p* = 0.589). However, the bilateral depth of the IJV on SBTC was larger than that on the bilateral CC level (*p* < 0.01). The MAPD and CSA of the IJV on the right CC were significantly larger than those of the other three measuring points (*p* < 0.01) followed by those on the left CC (*p* < 0.01). The smallest MAPD and CSA of the IJV were noted on the left SBTC (Figure 2).

**Table 2:** The anthropometric values of both internal jugular veins on the levels of cricoid cartilage and superior border of thyroid cartilage.

	**Depth (mm)**	**MAPD (mm)**	**CSA (mm^2^)**
RCC	8.8 ± 2.0	9.5 ± 2.1	96.7 ± 41.7
RSBTC	10.1 ± 2.3ab	7.5 ± 1.4ab	52.1 ± 19.7ab
LCC	8.7 ± 1.9	8.2 ± 1.9a	65.2 ± 23.4a
LSBTC	9.8 ± 2.2ab	6.9 ± 1.5abc	46.2 ± 17.2ab

*RCC, right cricoid cartilage; RSBTC, right superior border of thyroid cartilage; LCC, left cricoid cartilage; LSBTC, left superior border of thyroid cartilage; MAPD, maximal antero-posterior diameter; CSA, cross-sectional area*.

*a, compared to RCC, p < 0.01; b, compared to LCC, p < 0.01; c, compared to RSBTC, p < 0.01*.

To estimate the depth, MAPD, and CSA of bilateral IJV, the anthropometric and demographic values were analyzed to determine whether a correlation existed between them. No significant correlation was noted between these measured values and the characteristics of the studied pediatric patients. Pearson's *R* correlation coefficient and the level of significance are shown in Table 3. The most significant correlation was between the MAPD of the left CC and weight, and the R was merely 0.45.

**Table 3:** The correlation coefficients between anthropometric values of both internal jugular veins on two levels and demographic variables of the studied pediatric patients.

	**Depth**		**MAPD**		**CSA**		**Depth**		**MAPD**		**CSA**	
	***R***	***P***	***R***	***P***	***R***	***P***	***R***	***P***	***R***	***P***	***R***	***P***
	**RCC**						**RSBTC**					
Age	0.19	0.47	0.38	0.00	0.36	0.00	0.16	0.11	0.33	0.001	0.29	0.002
Size	0.25	0.11	0.33	0.001	0.28	0.003	0.23	0.18	0.31	0.001	0.25	0.011
Weight	0.39	0.00	0.29	0.002	0.24	0.014	0.37	0.00	0.28	0.004	0.21	0.03
BSA	0.33	0.001	0.32	0.001	0.27	0.006	0.30	0.002	0.30	0.002	0.24	0.14
	**LCC**						**LSBTC**					
Age	0.12	0.205	0.33	0.001	0.27	0.004	0.15	0.12	0.25	0.01	0.21	0.03
Size	0.13	0.175	0.35	0.000	0.34	0.000	0.17	0.09	0.32	0.001	0.29	0.002
Weight	0.24	0.014	0.45	0.000	0.37	0.000	0.26	0.006	0.37	0.000	0.37	0.000
BSA	0.19	0.045	0.41	0.000	0.36	0.000	0.22	0.024	0.35	0.000	0.34	0.000

*RCC, right cricoid cartilage; RSBTC, right superior border of thyroid cartilage; LCC, left cricoid cartilage; LSBTC, left superior border of thyroid cartilage; MAPD, maximal antero-posterior diameter; CSA, cross-sectional area*.

For further analysis, all children were classified based on their ages into the following categories: <12 months, between 12 and 72 months, and >72 months. Because only three children were <12 months old, Pearson's *R* correlation coefficient was calculated again in the other two age groups. However, no significant correlation was found among these demographic and anthropometric values. Thus, regression analysis had to be abandoned.

## Discussion

IJV is the most frequent approach of central venous catheterization in pediatric patients (2). However, the best IJV venipuncture point has not been decided because different operators have different experiences (10, 11). When assessing the relationships between subcutaneous depth, MAPD, and CSA of IJV and pediatric patients' characteristics, it is beneficial to make decisions at the moment of IJV cannulation, for example, selecting the appropriate access point and catheter diameter.

Unfortunately, no significant correlations between parameters of both IJVs and characteristics of the studied pediatric patients were found in this study. These results differed from those noted in previous studies (12, 13) due to much weaker correlations. For further exploration, 107 pediatric patients were classified on the basis of their ages. In the two groups of patients with ages between 12 and 72 months and older than 72 months, a significant correlation was still not demonstrated. In the study of Uzumcugil and Ekinci, no correlation was noted between antero-posterior diameter and weight in patients older than 45 weeks, which was consistent with our findings. All enrolled patients in the study were infants and neonates in Turkey (13). However, this correlation was demonstrated in a study of Spanish researchers (12), so we inferred that these distinctions might be caused by different races. The different measurement positions might be another factor that explains these different results.

Although many formulas were built on the basis of patients' age, weight, and height, which were convenient to guide pediatric IJV catheters, these formulas were not easy to remember and were unavailable in some conditions (14). Given different correlative coefficients in different studies, there is no guidance or consensus with respect to the direct calculation of IJV CSA or diameter based on patients' height and weight in the clinic. In addition, because the correlation was better for other vessels compared with the IJV (12) and ~2% anatomical variation of the IJV occurred, these factors increased the difficulty and uncertainty of estimating IJV features (15). Clinically, evaluating IJV traits and ambient anatomic structures using US devices is a quick process; thus, multiple regression analysis was abandoned in this study.

Among all measurements, the MAPD and CSA of the right IJV were the greatest followed by that of the left IJV. This finding was consistent with a previous study (16), which showed that the left IJV was slightly thinner and smaller in dimension than the right IJV. Regardless of the CC level or SBTC level, both the MAPD and CSA of the right IJV were greater than those of the left IJV, and the right IJV's CSA was more obvious. This information indicates that the right IJV is easier to use and more popular for venous access than the left IJV.

In 2015, Buch et al. (17) studied anatomic variations of the caliber of the IJV over its extracranial course with computer tomographic images. From C1 to C7, the CSA of the IJV increased gradually, and this tendency existed on the bilateral IJV. These results were demonstrated by the present study again, in which the IJV at the level of CC was bulkier than that at the level of SBTC. Because SBTC is approximately equal to transverse process of C4 and CC approximately equal to the transverse process of C6 (18, 19), the IJV on the level of CC was larger than that on the level of SBTC for both the right and left sides. In this study, the CSA disparity between the two levels was ~41–46%. Above the intracranial level of SBTC, the IJV might be much slimmer (17), which makes IJV access more difficult at the level of SBTC.

In this study, the phenomenon of IJV bifurcation was observed only at the level of bilateral SBTC but not at the level of CC (detailed data and images were not provided), which also increased the difficulty of IJV access at the SBTC level. In addition, the distance between SBTC and the mandibular angle in pediatric patients is very short, and such a cramped space is too limited for IJV puncture. If the IJV puncture point was moved down to the CC level, that is, to the level of the sixth cervical vertebra, then the operative distance would increase by at least 1 cm (20), as would the operative space. The US-assisted out-of-plane technique is available, and a special puncture transducer can be located perpendicular to the longitudinal axis of the patient's neck. If this space was still too limited for the out-of-plane technique, then the US evaluation and the location of the IJV might represent an effective substitution for the US guidance (21).

In this study, the CSA and MAPD of the IJV on the CC level were significantly increased compared with those of SBTC level. CSA was augmented by >40%, and MAPD increased by >1.3 mm. Although the CSA and MAPD of the IJV might continue to increase along its course to the supraclavicular area, these values did not change significantly from the CC to the jugular angle (22). On the basis of these varieties, we inferred that moving the IJV puncture point down beyond the CC level would not increase the success rate of IJV puncture given the similar vessel size. On the same course, the same lateral common carotid becomes thicker, and the arteriovenous overlap is relatively less at the mid-cervical level. Thus, an excessively low IJV access might be inappropriate. In addition to the pleural proximity at the low cervical level, the lower puncture point of the IJV should also increase pneumothorax complications due to inadvertent puncture of the parietal pleura or lung (23).

More importantly, the IJV and vertebral artery are closer as they move down the neck (22). Except for the vertebral artery, numerous branches of the subclavian artery approach the posterior wall of the IJV, which can be punctured at the lower neck level. Therefore, when IJV catheterization is performed, the puncture point is lower along the patient's neck, and the risk of vertebral artery puncture is greater (11, 24). The level of the CC approximates the level of the apex of the sternocleidomastoid muscle triangle, both of which are located on the midsection of the longitudinal axis of the neck. Therefore, IJV access on the CC level might offer more advantages than access to the upper and inferior neck segments (25).

In this study, IJV at the CC level was more superficial than that at the level of SBTC. This short subcutaneous depth might increase the chance of successful IJV access.

On the basis of the above evidence, the best location of IJV puncture was at the level of the right CC because the IJV was the most superficial and relatively bulkier. In addition, this appropriate anatomical site is close to the common artery and vertebral artery. These factors are beneficial for increasing the first success rate and reducing complications. If contraindications for right IJV access were noted, then the most favorable substitute location of IJV puncture was potentially on the left CC level.

## Limitations

In our study, there are still certain limitations to be considered when reviewing the results. First, the wide age range of the study patients, ranging from 6 months to 15 years, makes the interpretation of the study results difficult. In addition, only three patients were included in the age group under 1 year old. Thus, for more accurate interpretation of these results, Pearson's *R* correlation coefficient was calculated again with different age distributions. In future studies, we should focus on pediatric patients in a narrow age range. Second, there are apparently few differences in the absolute numbers of depths and MAPDs of bilateral IJVs, which can make readers doubt whether these differences have any significance in clinical situations, especially with the use of US for IJV puncture. However, the CSA of IJV differed by at least 50% (96.7 and 65.2 mm^2^), and this result obviously increased our confidence in IJV puncture in pediatric patients. In addition, more pediatric patients should be adopted in our future studies to provide a more accurate interpretation.

## Conclusion

Because the right IJV at the CC level is much bulkier and more superficial with an appropriate surrounding anatomic structure, this location is the optimal site for IJV puncture. If this location is unavailable, then the most appropriate alternative site is the left IJV on the same level. Meanwhile, a better correlation between the anthropometric parameters of IJV and children's biological characteristics was not observed. Because the target vein and its surrounding structures can be acquired easily with US assistance, it is unnecessary to estimate its anatomical traits using formulas. Their relationship should be further assessed in considerably larger-scale demographical studies in the future.

## Data Availability Statement

The original contributions presented in the study are included in the article/supplementary material, further inquiries can be directed to the corresponding author/s.

## Ethics Statement

The studies involving human participants were reviewed and approved by Ethical Committee of Sanbo Brain Hospital, Capital Medical University. Written informed consent to participate in this study was provided by the participants' legal guardian/next of kin.

## Author Contributions

JX and HW designed this clinical trial and wrote the primary article. YZhu and LZ measured values of internal jugular vein with ultrasound device and gave advices to statistics analysis. YZho and YP gave advices to this trail and English writing. All authors contributed to the article and approved the submitted version.

## Conflict of Interest

The authors declare that the research was conducted in the absence of any commercial or financial relationships that could be construed as a potential conflict of interest.

## Publisher's Note

All claims expressed in this article are solely those of the authors and do not necessarily represent those of their affiliated organizations, or those of the publisher, the editors and the reviewers. Any product that may be evaluated in this article, or claim that may be made by its manufacturer, is not guaranteed or endorsed by the publisher.
